# Fighting Pathogenic Bacteria on Two Fronts: Phages and Antibiotics as Combined Strategy

**DOI:** 10.3389/fcimb.2019.00022

**Published:** 2019-02-18

**Authors:** Thaysa Leite Tagliaferri, Mathias Jansen, Hans-Peter Horz

**Affiliations:** ^1^Institute of Medical Microbiology, RWTH Aachen University Hospital, Aachen, Germany; ^2^Department of Microbiology, Institute of Biological Sciences, Universidade Federal de Minas Gerais, Belo Horizonte, Brazil

**Keywords:** antibiotics, phages, phage therapy, phage and antibiotic combination, antimicrobial resistance, phage-antibiotic synergy (PAS), resistance evolution, ESKAPE

## Abstract

With the emerging threat of infections caused by multidrug resistant bacteria, phages have been reconsidered as an alternative for treating infections caused by tenacious pathogens. However, instead of replacing antibiotics, the combination of both types of antimicrobials can be superior over the use of single agents. Enhanced bacterial suppression, more efficient penetration into biofilms, and lowered chances for the emergence of phage resistance are the likely advantages of the combined strategy. While a number of studies have provided experimental evidence in support of this concept, negative interference between phages and antibiotics have been reported as well. Neutral effects have also been observed, but in those cases, combined approaches may still be important for at least hampering the development of resistance. In any case, the choice of phage type and antibiotic as well as their mixing ratios must be given careful consideration when deciding for a dual antibacterial approach. The most frequently tested bacterium for a combined antibacterial treatment has been *Pseudomonas aeruginosa*, but encouraging results have also been reported for *Escherichia coli, Staphylococcus aureus, Klebsiella pneumoniae, Acinetobacter baumannii, Enterococcus faecalis*, and *Burkholderia cepacia*. Given the immense play area of conceivable phage-antibiotic combinations and their potential excess value, it is time to recapitulate of what has been achieved so far. This review therefore gathers and compares the results from most relevant studies in order to help researchers and clinicians in their strategies to combat multidrug resistant bacteria. Special attention is given to the selected bacterial model organisms, the phage families and genera employed, and the experimental design and evaluation (e.g., *in vitro* vs. *in vivo* models, biofilm vs. planktonic culture experiments, order and frequency of administration etc.). The presented data may serve as a framework for directed further experimental approaches to ultimately achieve a resolute challenge of multidrug resistant bacteria based on traditional antibiotics and phages.

## Introduction

In the era of the increasing emergence of multi-drug resistant bacteria, a key question is currently being raised: do bacteriophages represent an alternative to antibiotics (Lin et al., 2017)? Numerous *in vitro* and *in vivo* studies using single or mixed phage types (phage cocktails) have been conducted over the years, however, a clear answer to this question has still not been provided (Nobrega et al., 2015). While in principle promising results have been reported, the establishment of phage therapy in modern Western medicine is a long and stony road, on which a number of hurdles have to be overcome (Pelfrene et al., 2016). Besides complicated regulatory issues and safety concerns, reluctance toward using phages for curing infectious diseases stems from prevailing skepticism about their true therapeutic efficiency, for example because of phage resistance evolution (Chanishvili, 2012). Because of such potential shortcomings of phages, the probably more adequate question would be whether the joint use of phages and antibiotics is the superior strategy for controlling bacterial pathogens. The expected benefit of such a dual approach might be the stronger bacterial suppression and the reduced bacterial capacity of developing phage and/or antibiotic resistance (Torres-Barceló and Hochberg, 2016). In fact, several studies investigating the combined benefit of phages and traditional antibiotics have provided encouraging results. For instance, it has been shown that sub-inhibitory concentrations of antibiotics can foster phage productivity and thus phage-mediated bacterial decline, a phenomenon termed phage-antibiotic synergy, or PAS (e.g., Comeau et al., 2007). This beneficial effect has been observed for some phage/antibiotic combinations (Ryan et al., 2012; Kamal and Dennis, 2015; Uchiyama et al., 2018), but not for others (Gelman et al., 2018; Torres-Barceló et al., 2018). A combined approach can also lead to the restoration of antibiotic sensitivity, for instance, in cases where the phage interacts with the bacterial drug efflux systems (Chan et al., 2016). Given the immense diversity of phages, there still exists a plethora of untapped phage-antibiotic combinations. Furthermore, positive interactions between any two antimicrobial agents may strongly depend on the treatment conditions (e.g., dosage, frequency, time points and order of administration etc.), which offers plenty of room for versatile experimentation. Knowledge and consideration of already tested phage/antibiotic “medleys,” whether proofing to be successful or not, may assist in the more directed elucidation of suitable combinations and conditions. This review therefore provides an overview of the most pertinent studies describing dual approaches, which may aid with the conception of optimized antibacterial strategies. We primarily focused on articles that were directed against selected members of the so-called ESKAPE-group, which includes *Enterococcus faecium, Staphylococcus aureus, Klebsiella pneumoniae, Acinetobacter baumannii, Pseudomonas aeruginosa*, and *Enterobacter* species (Rice, 2008) or directed against other opportunistic pathogens (such as *Escherichia coli, Enterococcus faecalis*, and *Burkholderia cepacia*). Depending on the magnitude of bacterial suppression, desirable positive interactions can be categorized as true synergism, additive effects, or as facilitation, the latter of which indicates that the combined approach is better than the best acting single agent, but worse than the sum of both antimicrobials acting independently (Chaudhry et al., 2017). Given that even facilitation is a desirable outcome, this has probably led to the tentatively broader use of the word “synergy,” as many studies use this term without further distinction for any improvement of the combined approach, as long as it is significant. Accordingly, unless otherwise stated, the term “synergy” in this paper refers to a combined antibacterial effect that is stronger compared to the best acting compound (phage or antibiotic) alone.

**Figure 1:**
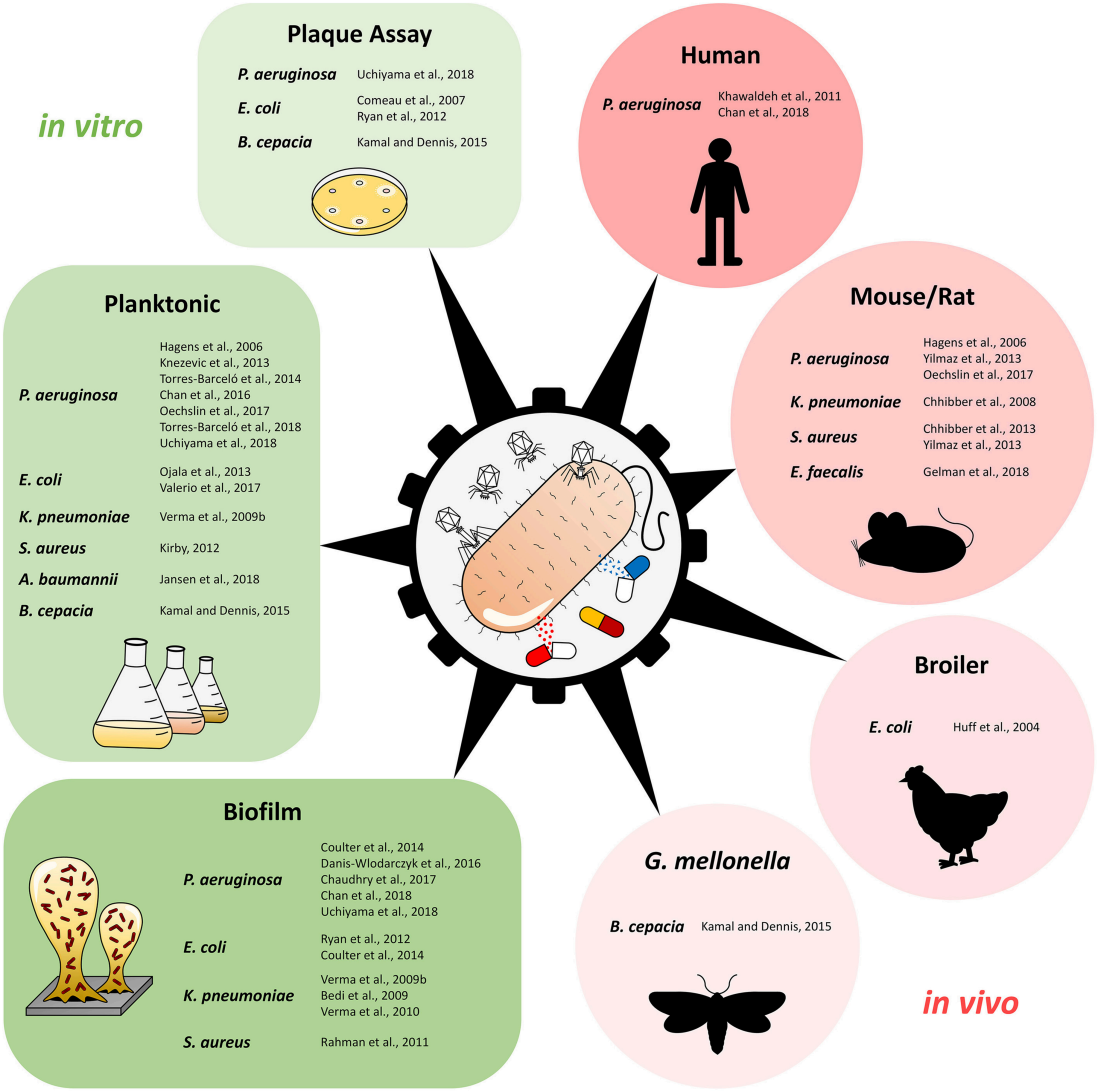
Overview of studies using phage-antibiotic combinations against pathogenic bacteria, separated by type of study and experimental design. Plaque Assay refers to studies that investigated phage-antibiotic synergy (PAS) based on plaque size on solid media.

Figure 1 and Table 1 provide an overview of the experimental approaches (i.e., *in vitro*/*in vivo* studies, planktonic culture/biofilm studies etc.). Table 1 includes also additional information about the selection of phage genus, antimicrobial agents, and the potential synergistic combinations. Further details, e.g., antibiotic and phage dosage used in each combination are listed in the Supplementary Table S1.

**Table 1:** Overview of phage-antibiotic combinations tested against human pathogenic bacteria^Δ^.

**Reference**	**Phage name (*Family*)**	**Phage genus^**+**^**	**Antibiotic classes**					
			**β-L**	**AG**	**FQ**	**PM**	**TC**	**OT**
***Pseudomonas aeruginosa***								
**Chaudhry et al. (****2017****)**	NP1 (*S*) + NP3 (*M*)	*NP1Virus* / n.s.l.	CAZ	TOB/GEN	CIP	CST		
**Danis-Wlodarczyk et al. (****2016****)**	KTN4 (*M*)	*phiKZ-like-virus*				CST		
**Coulter et al. (****2014****)**	PB-1 (*M*)	*Pbunavirus*^1^		TOB*				
**Torres-Barceló et al. (****2014****)**	LUZ7 (*P*)	*N4-like virus*^2^		STR				
**Torres-Barceló et al. (****2018****)**	LKD16 (*P*)	*Phikmvvirus*^1^	CAZ*		CIP*			ERY*
	LUZ7 (*P*)	*N4-like virus*^2^	CAZ*		CIP*			ERY*
	14/1 (*M*)	*Pbunavirus*^1^	CAZ*		CIP*			ERY*
	EL (*M*)	*Elvirus*^1^	CAZ*		CIP*			ERY*
**Uchiyama et al. (****2018****)**	KPP21 (*P*)	*N4-like virus*^2^	FEP/CZO/CFP/CFP+SUL/ CAZ/CTX/CPD/MOX/FMX/CTM/CMZ/PIP/MEM/IPM/ATM	GEN/TOB/AMK	CIP/LVX	CST	MIN	FOF/CHL/SXT
	KPP22 (*M*)	*Pbunavirus*	FEP/CZO/CFP/CFP+SUL/CAZ/CTX/CPD/MOX/FMX/CTM/CMZ/PIP/MEM/IPM/ATM	GEN/TOB/AMK	CIP/LVX	CST	MIN	FOF/CHL/SXT
	KPP23 (*S*)	n.s.l.	FEP/CZO/CFP/CFP+SUL/CAZ/CTX/CPD/MOX/FMX/CTM/CMZ/PIP/MEM/IPM/ATM	GEN/TOB/AMK	CIP/LVX	CST	MIN	FOF/CHL/SXT
	KPP25 (*P*)	*Kpp25virus*	FEP/CZO/CFP/CFP+SUL/ CAZ/CTX/CPD/MOX/ FMX/CTM/CMZ/PIP/MEM /IPM/ATM	GEN/TOB/AMK	CIP/LVX	CST	MIN	FOF/CHL/SXT
**Knezevic et al**. (**2013****)**	δ (*P*)	n.s.l.	CRO	GEN	CIP	PMB		
	δ-1 (*S*)	n.s.l.	CRO	GEN	CIP	PMB		
	001A (*S*)	n.s.l.	CRO	GEN	CIP	PMB		
**Chan et al. (****2016****)**	OMKO1 (*M*)	*phiKZ-like-virus*	CAZ		CIP		TET	ERY
**Chan et al. (2018)**	OMKO1 (*M*)	*phiKZ-like-virus*	CAZ		CIP			
**Khawaldeh et al. (****2011****)**	Pyophage cocktail	n.s.l.	MEM			CST		
**Oechslin et al. (****2017****)**	PP1131 cocktail	n.s.l.	MEM*		CIP*			
**Yilmaz et al. (****2013****)**	PAT14 (*P*)	n.s.l.	IPM+CIL	AMK				
**Hagens et al. (****2006****)**	Pf3 (*I*)	*Inovirus*^3^	CAR	GEN			TET	CHL
	Pf1 (*I*)	*Inovirus*^3^	CAR	GEN			TET	CHL
***Escherichia coli***								
**Comeau et al. (****2007****)**	Φ MFP (*S*)	n.s.l.	CTX/ATM/CFM/CRO/CAZ	GEN			TET	
	RB32 (*M*)	n.s.l.	CTX					
	RB33 (*M*)	n.s.l.	CTX					
	T3 (*P*)	*T7virus*^1^	CTX					
	T7 (*P*)	*T7virus*^1^	CTX					
	T4 (*M*)	*T4virus*^1^	**CTX**/**PIP**/**AMP**/**TIC**		**NAL**			**MTC**
**Ryan et al. (****2012****)**	T4 (*M*)	*T4virus*^1^	CTX					
**Coulter et al. (****2014****)**	T4 (*M*)	*T4virus*^1^		TOB*				
**Valério et al. (****2017****)**	ECA2 (*P*)	n.s.l.	AMP/PIP	KAN	CIP*		TET	CHL
**Ojala et al. (****2013****)**	PRD1 (*T*)	*Tectivirus*^1^		KAN*				RIF*
**Huff et al. (****2004****)**	SPR02 + DAF6	n.s.l.			ENR			
***Staphylococcus aureus***								
**Rahman et al. (****2011****)**	SAP-26 (*S*)	*Phietavirus*^1^						VAN/RIF/AZM
**Kirby (****2012****)**	SA5 (*M*)	*Kayvirus*^1^		GEN*				
**Yilmaz et al. (****2013****)**	Sb-1 (*M*)	*Kayvirus*^1^						**TEC**
**Chhibber et al. (****2013****)**	MR-10 (*M*)	n.s.l.						LZD
***Klebsiella pneumoniae***								
**Verma et al. (****2009b****)** and **(****2010****)**	KPO1K2 (*P*)	*T7-like virus*^4^			CIP*			
**Bedi et al. (****2009****)**	n.s.l.	n.s.l.	AMX					
**Chhibber et al. (****2008****)**	SS (*P*)	n.s.l.		AMK				
***Acinetobacter baumannii***								
**Jansen et al. (****2018****)**	KARL-1 (*M*)	*T4-like virus*	MEM*		CIP	CST		
***Enterococcus faecalis***								
**Gelman et al. (****2018****)**	EFDG1+EFLK1 (*M*)	n.s.l.	AMP*	
***Burkholderia cepacia***								
**Kamal and Dennis (****2015****)**	KS12 (*M*)	n.s.l.	AMP/CAZ/PIP/MEM	KAN	CIP/LVX		TET/MIN	
	KS14 (*M*)	*P2-like-virus*	AMP/CAZ/PIP/MEM	KAN	CIP/LVX		TET/MIN	

Δ *Experimental studies appear in the same order as in the main text. Antibiotics in green indicate positive interaction (enhanced bacterial suppression or PAS) with the respective phage; antibiotics in black indicate that positive interactions with respective phage were not observed; Stars behind the antibiotics mark those studies in which resistance evolution was investigated; green stars indicate that the combined approach reduced the emergence of resistant cells; black stars indicate that the emergence of resistant cells was not reduced or the sensitivity level was maintained with the combined approach, respectively. Gray scale: in vitro studies; light red scale: in vivo studies; dark red scale: human case reports*.

+ *Phage genus information was provided by the respective references in the table (left column), except for cases with superscript numbers: ^1^Mihara et al. (2016); ^2^Shen et al. (2016); ^3^Holland et al. (2006); ^4^Verma et al. (2009a), which do not represent studies about phage/antibiotic combinations, but provide information about the phage genus*.

*(M), Myoviridae; (I), Inoviridae; (P), Podoviridae; (S), Siphoviridae; (T), Tectiviridae; n.s.l.: genus not specified in literature; β-L, Beta-Lactam antibiotics/Beta-Lactamase inhibitors; AG, Aminoglycosides; FQ, Fluoroquinolones; PM, Polymyxins; TC, Tetracyclines; OT, Others; AMK, amikacin; AMP, ampicillin; AMX, amoxicillin; ATM, aztreonam; AZM, azithromycin; CAR, carbenicillin; CAZ, ceftazidime; CFM, cefixime; CFP, cefoperazone; CHL, chloramphenicol; CIL, cilastatin; CIP, ciprofloxacin; CMZ, cefmetazole; CPD, cefpodoxime; CRO, ceftriaxone; CST, colistin; CTM, cefotiam; CTX, cefotaxime; CZO, cefozopran; ENR, enrofloxacin; ERY, erythromycin; FEP, cefepime; FMX, flomoxef; FOF, fosfomycin; GEN, gentamicin; IPM, imipenem; KAN, kanamycin; LVX, levofloxacin; LZD, linezolid; MEM, meropenem; MIN, minocycline; MOX, moxalactam (latamoxef); MTC, mitomycin C; NAL, nalidixic acid; PIP, piperacillin; PMB, polymyxin B; RIF, rifampicin; STR, streptomycin; SUL, sulbactam; SXT, trimethoprim/sulfamethoxazole; TEC, teicoplanin; TET, tetracycline; TIC, ticarcillin; TOB, tobramycin; VAN, vancomycin*.

## Pseudomonas aeruginosa

So far, the majority of phage/antibiotic studies have focused on *P. aeruginosa*, apparently because of its important clinical impact as opportunistic pathogen, which is often involved in cystic fibrosis, burn infections, hospital-acquired pneumonia, urinary tract infections, among others (Sousa and Pereira, 2014). *P. aeruginosa* has a strong colonization capacity on biotic and abiotic surfaces and persists against a wide range of antimicrobials (Kung et al., 2010; Alshalchi and Anderson, 2015). Biofilms and planktonic cultures of *P. aeruginosa* were the major target of many studies using mostly the strain PA01 or other reference strains, such as PA14, CHA, and PAK (De Soyza et al., 2013). Furthermore, a few case reports as well as some *in vivo* studies based on mice or rats as model organisms have been published.

Chaudhry et al. treated a 48-h biofilm of PA14 with the two phages NP1 (*Siphoviridae, NP1Virus*) and NP3 (*Myoviridae*) together or both in combination with five antibiotics (Chaudhry et al., 2017). Each antimicrobial alone showed only moderate anti-biofilm efficacy, however, when applied simultaneously, true synergistic effects *sensu stricto* were observed between phages and ceftazidime at 1x MIC and 8x MIC and for ciprofloxacin at 1x MIC. An improved effect by way of facilitation was also achieved for ciprofloxacin at 8x MIC and for tobramycin at 1x MIC, but interestingly not at 8x MIC (Chaudhry et al., 2017). These findings indicate the dose dependency of simultaneous applications with higher antibiotic concentrations likely removing the minimum bacterial density required for optimal phage replication. No improvement was observed with colistin and gentamicin, the latter of which is somewhat surprising, given that this antibiotic belongs to the same class as tobramycin. The therapeutic outcome differed with time-delayed use of phages and antibiotics. The addition of tobramycin or gentamicin 24 h after phage application led to a significant synergistic effect. Conversely, successive addition of ciprofloxacin or ceftazidime did not lead to a better outcome compared to the simultaneous application. Hence, critical to a successful combined application is the dosage and the time point of antibiotic addition. Variations in the phage dosage may also impact the antibacterial outcome, which was, however not further evaluated in this study.

Anti-biofilm activity but no synergistic effect was seen when the giant phage KTN4 (*Myoviridae, phiKZ-like-virus*) was combined with colistin against strain PAO1 grown for 24, 48, and 72 h *in vitro* (Danis-Wlodarczyk et al., 2016). Both antimicrobials alone achieved already a significant biofilm reduction, and as a possible explanation, the authors presume that colistin could limit phage propagation as it destabilizes the cell membrane. Conversely, phage KTN4 recognizes IV-type pili as receptor and therefore does not interfere positively or negatively with colistin activity (Danis-Wlodarczyk et al., 2016).

Likewise, a 48-h-old biofilm of PAO1 could not be stronger reduced with phage PB-1 (*Myoviridae, Pbunavirus*,) and tobramycin together (Coulter et al., 2014). However, the combination resulted in a significant decrease in the emergence of antibiotic and phage resistant bacterial cells. Thus, the treatment of biofilms using the dual approach is clearly warranted, despite an apparent lack of a stronger anti-biofilm capacity.

Irrespective of this, the challenge of planktonic cultures of PA01 generally led to more promising results. Torres-Barceló et al. applied phage LUZ7 (*Podoviridae, N4-like virus*) in conjunction with streptomycin which reduced the bacterial cell density significantly stronger than each single treatment (Torres-Barceló et al., 2014). Notably, the time point of antibiotic addition mattered (i.e., streptomycin administration 12 h after phage application achieved higher bacterial suppression than after 24 h), the result of which was independent from the streptomycin dosages. Hence, corroborating the findings of Chaudhry et al., there exists a specific time window during which the supplementary delivery of the antibiotic leads to optimal results (Chaudhry et al., 2017). Realization of the ideal time period may be the key for successful future applications.

However, larger time scales must also be taken into consideration. For instance, long-term effects over several days of four unrelated phages were investigated in addition with sub-inhibitory concentrations of ceftazidime, ciprofloxacin, and erythromycin applied against the strain PAO1 (Torres-Barceló et al., 2018). The phages used were LKD16 (*Podoviridae, Phikmvvirus*), LUZ7 (*Podoviridae, N4-like virus*), 14/1 (*Myoviridae, Pbunavirus*), and EL (*Myoviridae, Elvirus*). Except for ciprofloxacin, phage density initially decreased in most tested antibiotic-phage combinations, which is somewhat opposing the phenomenon of PAS. Even phage virulence, defined as the capacity to inhibit ancestral bacterial density, was reduced in the co-presence of antibiotics. Those negative effects on the phages were not observed later on (i.e., after 8 days), indicating that phages had adapted to the antibiotic-containing environment. Despite of this, combination treatments stronger controlled bacterial density and particularly ciprofloxacin limited antibiotic resistance evolution. Thus, this study principally encourages combination strategies, but also points at the need for investigations at a prolonged time scale (i.e., several days, comparable to the time window of an infectious disease under treatment) in order to account for—and understand—the clinical importance of evolutionary adaptations during phage therapy.

In an attempt to systematically identify well-working phage/antibiotic combinations, Uchiyama et al. tested PAS, using four unrelated phages and 25 antibiotics against strain PAO1 and five clinical isolates of *P. aeruginosa* (Uchiyama et al., 2018). The four phages were KPP21 (*Podoviridae, N4-like virus*), KPP22 (*Myoviridae, Pbunavirus*), KPP23 (*Siphoviridae*), and KPP25 (*Podoviridae, Kpp25virus*). While no PAS was observed between KPP25 and any of the antibiotics, the other three phages exhibited PAS with 5, 13, and 3 antibiotics, respectively. Involved in PAS were predominantly cell wall synthesis inhibiting antibiotics including the anti-*Pseudomonas* drugs ceftazidime and piperacillin. PAS was further confirmed for the best scoring phage KPP22 based on time-kill curves and biofilm assays, along with the testing of additional clinical isolates. The study shows that PAS can be observed quite frequently, although the selection of the phage type seems to be a crucial factor. It remains to be demonstrated though, whether PAS automatically qualifies successfully tested combinations for *in vivo* applications. Conversely, it is unclear whether or not those phage/antibiotic pairs, displaying no PAS, are *de facto* unsuitable as potential treatment option. This is an unexplored field and requires further investigation.

The suppressive effect against reference strains other than PA01 was assessed by Knezevic et al., using three unrelated phages (Knezevic et al., 2013). Phages δ (*Podoviridae*), δ-1, and 001A (both *Siphoviridae*) were used against their individual hosts, i.e., PA-4U, ATCC 9027, and PA-M2, respectively, in conjunction with sub-inhibitory concentrations of ciprofloxacin, ceftriaxone, gentamicin, or polymyxin B. Only with ceftriaxone an enhanced reduction of planktonic cultures was observed. In addition, a synergistic effect—*sensu stricto*—occurred only with the combination of phage δ-1. This study again shows, that not every phage-antibiotic combination supresses bacteria stronger. The precise mode of antibiotic action (e.g., cell elongation) and the molecular base of phage/host interactions are important determinants of success. Nonetheless, the emergence of resistant variants might be reduced also with less successful combinations. This latter issue should therefore always be investigated, whenever combination approaches are evaluated.

Encouragement for a clinical approach was fuelled by the *in vitro* observation that the lytic phage OMKO1 (*Myoviridae, phiKZ-like-virus*) led to the re-sensitization to several antibiotics of eight *P. aeruginosa* strains including PAO1 (Chan et al., 2016). This phage binds to the outer membrane porin M, which belongs to certain efflux systems responsible for antibiotic resistance. Consequently, efflux pump mechanisms are severely affected by such a phage attack. Apparently, the attempt to evade the phage infection requires mutation adaptation that represents a genetic trade-off between phage resistance and antibiotic sensitivity. Chan et al. (2018) subsequently assessed the efficiency of the combined treatment of phage OMKO1 and antibiotic in a patient with prosthetic vascular graft infection in the aorta artery (Chan et al., 2018). Prior to the human application, the clinical isolate of the patient was tested in an *in vitro* biofilm assay. Both, ceftazidime and ciprofloxacin at 2x MIC were not sufficient to eliminate a 72-h old biofilm. In contrast, phage OMKO1 as single agent significantly reduced mean cell densities, but the biofilm was not stronger reduced by further adding antibiotics. However, since no antagonism was observed and given the aforementioned *in vitro* results (Chan et al., 2016), the patient was treated with a combined phage/antibiotic approach. In fact, following a single application of phage OMKO1 and ceftazidime, the infection appeared to resolve with no signs of recurrence (Chan et al., 2018). However, part of the graft was excised 4 weeks after the treatment due to aortic perforation. Hence, at this point it remains unclear whether the treatment success was due to this intervention, the phage activity alone, or its combination with ceftazidime (Chan et al., 2018).

In another case report Khawaldeh et al. described the successful use of six lytic *P. aeruginosa* phages combined at equal amounts into a Pyophage cocktail (Villarroel et al., 2017), as adjunctive therapy (Khawaldeh et al., 2011). While antibiotics alone failed to cure a recurrent bladder infection in a 67-year-old woman, the combination of the phage cocktail with meropenem and colistin led to symptomatic relief and reduction of the bacterial load, when applying the cocktail every 12 h for 10 days. Interestingly, a decrease in viable bacterial counts was already observed before starting the time-delayed antibiotic therapy which commenced on the sixth day of phage therapy. From then on, the bacterial count further decreased (day 7) until no viable bacteria could be detected anymore from the eighth day. This case report is encouraging, because the treatment was well-tolerated by the patient and because the beneficial effect of successive application of different antimicrobials agrees well with the aforementioned *in vitro* observations (Torres-Barceló et al., 2014).

Oechslin et al. (2017) evaluated the anti-*Pseudomonas* phage cocktail PP1131 containing 12 phages in combination with either 2.5x MIC of ciprofloxacin or meropenem against the *P. aeruginosa* strain CHA *in vitro* (Oechslin et al., 2017). With both antibiotics a significant synergistic effect with PP1131 was observed, and emerging phage-resistant subpopulations could be prevented by co-addition of the antibiotics. This positive effect could subsequently be confirmed when treating an experimental endocarditis model in rats. While a single application of the phage cocktail or ciprofloxacin was equally effective in reducing the bacterial load, a significant synergistic effect was achieved when both antimicrobial agents were jointly used. In this case, 64% of tested rats could be successfully treated. Hence, the combination approach proved to be meaningful in this infection model. However, *P. aeruginosa*-associated endocarditis is relatively rare in humans, which means that it would be interesting to know whether or not more common bacterial causes of this heart disease (e.g., staphylococci or streptococci) can also be treated with phage/antibiotic combinations. To our knowledge this has not been investigated so far.

Rats were also selected for an implant-related infection model using clinical isolates of *P. aeruginosa* followed by a subsequent treatment with phage vB_PsaP PAT14 (*Podoviridae*) in combination with imipenem/cilastatin, and amikacin (Yilmaz et al., 2013). The phage was administered through the skin, directly into the medullary canal, once a day for 3 consecutive days. The antibiotics were applied intraperitoneally once a day for 14 days. While the number of colony-forming units could be significantly stronger reduced during the combination therapy compared to the control group and the two groups receiving phage or antibiotics only, no significant difference was observed in the final biofilm thickness across the different treatments. Besides a too short follow-up time, failure to reduce the biofilm was ascribed to the selected phage, which apparently was not effective enough for biofilm degradation.

In contrast to the aforementioned studies, which focused on lytic phages, Hagens et al. examined the impact of the filamentous phages Pf3 and Pf1 (*Inoviridae, Inovirus*) on the dosage of certain antimicrobials required to inhibit the growth of *P. aeruginosa* strains PAO1 and PAK, respectively (Hagens et al., 2006). Up to 10-fold lower concentrations of antibiotics were needed in the presence of the filamentous phages. Even re-sensitization, despite the carriage of a plasmid containing resistance genes against antibiotics (including gentamicin and tetracycline), could be achieved with those phages. Finally, the authors evaluated the therapeutic effect of gentamicin and phage Pf1 in an intraperitoneal infection mouse model with the PAK strain. As a result, 16 out of 20 mice survived the 7-day observation period, whereas the control groups died within 48 h. As a major mechanism, it is plausible to assume that the extrusion of filamentous phage progenies may weaken the antibiotic-barrier function of the outer membrane of gram-negative bacteria. Thus, the effect of filamentous phages merits further investigation as potential complementation of antibiotics against multi-drug resistant bacteria.

## Escherichia coli

Although generally being a commensal in the gastrointestinal tract, *E. coli* is also recognized for intestinal and extra intestinal disorders such as diarrhea, colitis, urinary tract infections, bacteremia, as well as sepsis (Blount, 2015; Vila et al., 2016). It has been estimated that until 2050 more than 3 million people will die due to infections caused by multi-drug resistant *E. coli* (O'Neil, 2016).

The term PAS had first been introduced by Comeau et al., based on an uropathogenic strain of *E. coli* (MFP) and a lytic siphovirus, co-isolated from a patient with urinary tract infection (Comeau et al., 2007). It was found that this lytic phage (ΦMFP) benefits from sub-lethal doses of beta-lactams leading to a higher burst size and thus to increased plaques on agar plates (Comeau et al., 2007). This effect was not observed with tetracycline and gentamicin as well as phage ΦMFP. With phage T4 (*Myoviridae, T4virus*) PAS was also detected using quinolone and mitomycin C as well as further beta-lactam antibiotics. Cefotaxime also favored PAS with phages RB32 and RB33 (both *Myoviridae*) against strain MFP. In addition, while PAS could also be demonstrated for the *E. coli* strain AS19 with the phages T4, T3 (*Podoviridae, T7virus*), and T7 (*Podoviridae, T7virus*) the authors found that PAS occurred independently of the bacterial SOS system and was rather due to cellular filamentation upon exposure to respective antibiotics. Hence, there is a wide distribution of PAS across unrelated phages, however, as stated above, the true value of this phenomenon for phage therapy remains to be elucidated.

Complementary results were observed by Ryan et al., who observed PAS with phage T4 (*Myoviridae, T4virus*) and distinct concentrations of cefotaxime against *E. coli* strain ATCC 11303 (Ryan et al., 2012). Besides an increased burst size, along with a reduced latent period of T4, the dual combination had a significantly stronger anti-biofilm capacity compared to the single biofilm treatments. Also, with increasing phage titers, decreasing levels of cefotaxime were needed for the eradication of a 24-h biofilm. Thus, this study demonstrated for the first time that PAS affected bacterial biofilms. Furthermore, decreasing the effective therapeutic level of an antibiotic with phages could be a beneficial strategy to minimize adverse side effects of the antibiotics *in vivo*. By using the same strain and the same phage—this time combined with tobramycin—a 48-h biofilm could nearly be completely eradicated after 24 h of exposure, in contrast to the single treatments (Coulter et al., 2014). Moreover, the combined strategy prevented the occurrence of antibiotic- and phage-resistant cells by 99 and 39%, respectively.

Sub-lethal doses favored synergistic effects between the phage ECA2 (*Podoviridae*) and ciprofloxacin against the *E. coli* strain ATCC 13706 (Valério et al., 2017). Interestingly, this effect was not observed with higher antibiotic concentrations. No synergy was observed with the bacteriostatic antibiotics tetracycline and chloramphenicol and with antibiotics against which the strain was *a priori* resistant, i.e., piperacillin, ampicillin, and kanamycin. In agreement with the synergistic result, the authors also found a lower frequency of resistant mutants when the sub-inhibitory concentration of ciprofloxacin was used. Finally, using identical treatment conditions, the synergistic effect of the dual treatment could be confirmed based on the *in vitro*-simulation of an *E. coli*-driven urinary tract infection with real urine samples.

An elegant and somewhat different approach was described by Ojala et al. Instead of the attempt to maximize bacterial suppression with combined antimicrobials, their goal was the prevention of the spread of drug resistance genes via conjugative plasmids (Ojala et al., 2013). To this end, the phage PRD1 (*Tectiviridae, Tectivirus*), which adsorbs to receptors encoded by conjugative plasmids, was used. The presence of this phage shifted the selective pressure toward *E. coli* strains that were plasmid free and thus became sensitive to certain antibiotics. This positive effect, although less strongly pronounced, was also seen with the co-presence of kanamycin or rifampicin, suggesting that a combination approach is suitable for obtaining plasmid-free cells. Although not performed with clinical strains but with the two reference strains (i.e., *E. coli* K-12 strains JE2571 and HMS174), the study provides proof-of principle that conjugative-plasmid dependent phages might represent a valuable complementation of antimicrobial therapies. Notably, the host range of phage PRD1 is determined by the host range of suitable conjugative plasmids, which means that this phage can be applied against many other gram-negative species as well (Ojala et al., 2013). However, more research is needed, especially for assessing the functionality of this system against clinical isolates and under more complex *in vivo* conditions.

By way of an example of a successfully tested *in vivo* model, Huff et al. treated broiler chicken simultaneously with enrofloxacin (a fluoroquinolone antibiotic used for the treatment of domestic animals), with a mixture of the phages SPR02 and DAF6 and rescued all individuals that were experimentally infected with an avian pathogenic *E. coli* isolate (Huff et al., 2004). This result was in clear contrast to the single application of phages or enrofloxacin, which led to mortality rates of 15 and 3%, respectively, which were, however, significantly lower than those of untreated chicken (i.e., 68%). Hence, this study demonstrates that phage/antibiotic approaches may have a practical and exploitable value for poultry and animal production systems.

## Staphylococcus aureus

Although present in about 30% of population as a commensal bacterium, *S. aureus* is a leading cause of bacteraemia as well as infective endocarditis. It is also responsible for osteoarticular-, skin-, and device-related infections, among others (Tong et al., 2015; Oliveira et al., 2018). Importantly, MRSA is responsible for 13% up to 74% of all *S. aureus* infections worldwide, with different incidences around the world (Hassoun et al., 2017). Treatment options are limited for MRSA (Lee et al., 2018) and usually comprise the administration of linezolid, vancomycin, or daptomycin when the infection is invasive (Wunderink et al., 2012; Choo and Chambers, 2016). Therefore, several MRSA preventive strategies are under consideration (Lee et al., 2018), one of which could be the combination of phages and antibiotics.

In order to eradicate the biofilm of the clinical isolate *S. aureus* D43-a, phage SAP-26 (*Siphoviridae, Phietavirus*) was administered simultaneously with azithromycin, vancomycin, or rifampicin (Rahman et al., 2011). A synergistic effect was observed during treatment of the 24-h-old biofilm with SAP-26 and rifampicin leading to around 35% of surviving cells, while phage combinations with azithromycin or vancomycin revealed survival rates of about 40% and 60%, respectively. With phage alone, 72% of the bacteria survived, whereas the survival rate with single rifampicin, azithromycin, or vancomycin application was 60, 75, and 83%, respectively. Biofilm eradication was also demonstrated by field emission scanning electron microscopy, which identified only few bacterial cells, most of which with irregular morphology after combined therapy. Thus, this study showed for the first time that an *S. aureus* biofilm can efficiently be reduced by the use of an appropriate mixture of phage and antibiotic, in this case rifampicin.

By using a continuous culture system, the dual treatment of gentamicin and phage SA5 (*Myoviridae, Kayvirus*) was tested against the clinical isolate PS80 (Kirby, 2012). In fact, the combination was more efficacious than single therapies after 72 h of treatment. The synergistic effect was explained by gentamicin leading *S. aureus* cells to assume an aggregate phenotype. And although this phenotype eases biofilm formation (as an attempt to evade antibiotic activity), it is also more susceptible to the phage attack, resulting ultimately in lower cell densities (Kirby, 2012). Even more, no phage resistant cells were identified in the dual approach as opposed to the phage-only treatment. Notably, aggregate formation upon antibiotic exposure has frequently been reported for other strains and species (Kirby, 2012), indicating that this antimicrobial combination may be of broader suitability.

Yilmaz et al. evaluated the therapeutic potential of Phage Sb-1 (*Myoviridae, Kayvirus*) and teicoplanin in a rat tibiae infection model induced by a clinical isolate of MRSA (Yilmaz et al., 2013). The antibiotic was applied intraperitoneally once a day for 2 weeks, while the phage was administered through the skin, directly into the medullary canal, once a day for 3 consecutive days. This treatment resulted in more than 3-fold decrease of colony-forming units compared to the single application of the antibiotic and more than 6-fold decrease compared to the application of phage Sb-1 alone. Moreover, the development of a biofilm was only prevented with the combination therapy. For this reason, local phage application as adjunct to antibiotic therapy against MRSA holds great potential for use in orthopedic surgery.

Comparably promising results were obtained by treatment of diabetic mice with MRSA-induced hindpaw foot infections (*S. aureus* strain ATCC 43300). Treatment was performed with a local administration of phage MR-10 (*Myoviridae*) and a simultaneous oral application of linezolid (Chhibber et al., 2013). When assessed after 1, 3, and 5 days, the combination led to the strongest reduction of the bacterial load compared to mono-treatments, which was also verified by a stronger decline of clinical signs, such as lesion score, foot myeloperoxidase activity, and histopathology. Measurements after 7, 9, and 12 days of treatment revealed entire absence of bacteria in the combination treatment, but also in the monotherapy groups. Although the bacterial load did not differ significantly among the different treatment groups, the fact that the overall tissue healing was expedited argues for the combined treatment approach for preventing foot infections with MRSA. Clearly, diabetic foot infections are polymicrobial (Jneid et al., 2018), however, MRSA is highly prevalent and difficult to treat in diabetes patients worldwide. Using phage MR-10 with its reported host range >90% of tested clinical *S. aureus* isolates in combination with antibiotics could therefore at least mitigate the overall complications associated with such infections (Chhibber et al., 2013).

## Klebsiella pneumoniae

*K. pneumoniae* causes serious infections, especially in immunocompromised individuals, including pneumonia, bacteremia, or meningitis (Decré et al., 2011; Paczosa and Mecsas, 2016). However, some hyper-virulent *K. pneumoniae* strains have been reported to affect also healthy individuals (Paczosa and Mecsas, 2016). Allied to this, the ability of this bacterium to resist against a considerable number of antimicrobials asks for alternative strategies to treat *K. pneumoniae* infections.

For instance, the anti-biofilm effect of the combination of the phage KPO1K2 (*Podoviridae, T7*-*like virus*) and ciprofloxacin did not lead to a significant difference compared to single administrations applied on a 12-h-old biofilm of *K. pneumoniae* (Verma et al., 2009b). However, there was no negative interference and the frequency of emerging antibiotic or phage resistant cells was significantly lower with the combined approach. Unfortunately, with continued age of the biofilm, the anti-biofilm efficiency of either compound alone and together dropped markedly (Verma et al., 2010). When switching to amoxicillin as the antibiotic complement to the phage, the outcome of the dual approach scored better with minor statistical significance, indicating that beta-lactams are the preferable partner to phages against this species (Bedi et al., 2009). The same group also exploited the combined therapy to treat an experimental lobar pneumonia induced by *K. pneumoniae* B5055 in a mouse model (Chhibber et al., 2008). To this end, an intranasal injection of the podovirus SS (*Podoviridae*) was added together with amikacin. Again, the authors reported no additional advantage with the combined approach, however, they hinted at the different antimicrobial actions of both compounds, which should minimize the emergence of resistance. Unfortunately, this was not further investigated in this study. Given that only one reference strain and only phages from the *Podoviridae* family were tested against *K. pneumoniae* so far, a gallery of combinations still awaits to be explored against this pathogen.

## Acinetobacter baumannii

*A. baumannii* is responsible for several outbreaks worldwide (Dijkshoorn et al., 2007) causing a wide spectrum of infections including bacteremia, meningitis, pneumoniae as well as wound- and urinary tract infections (Peleg et al., 2008). Besides a high tolerance against harsh environmental conditions such as desiccation, UV, detergents, and disinfectants, intrinsic and acquired antibiotic resistance mechanisms constitute a major obstacle for controlling this nosocomial pathogen (Wendt et al., 1997; Wisplinghoff et al., 2007; Peleg et al., 2008). Consequently, strong interest in alternative antibacterial strategies exists also for *A. baumannii*. Nevertheless, we are aware of only one study, in which the combined use of antibiotics and phages was investigated.

Jansen et al. tested phage vB_AbaM-KARL-1 (*Myoviridae, T4-like virus*) in combination with each of the three antibiotics, meropenem, ciprofloxacin, and colistin against multi-drug resistant clinical isolates (Jansen et al., 2018). Although a complete clearance of planktonic *A. baumannii* cultures was achieved at a phage MOI of 10^−1^ and meropenem, the extent of additional bacterial suppression was most strongly pronounced when the phage titer was very low (i.e., MOI of 10^−7^). Likewise, significant stronger antibacterial effects were observed with colistin using the phage at an MOI of 10^−7^. Apparently, at higher phage titers, KARL-1 is already very effective with only little improvement by the co-addition of antibiotics. However, the lack of antibacterial efficiency due to a low amount of phages could be overcome by the addition of meropenem or colistin. Such an effect was not observed with ciprofloxacin. The authors also reported that the emergence of phage resistant variants could at least be hampered with the co-addition of meropenem. Whether or not the development of resistant variants could also be delayed with the other two antibiotics was not further investigated in this study.

## Enterococcus faecalis

*E. faecalis* is well-known as opportunistic pathogen related to nosocomial infections, endocarditis, and endodontic infections, among others (Fisher and Phillips, 2009; Muller et al., 2015; Madsen et al., 2017). The genetic plasticity of this species allowed it to succeed in the healthcare environment and the high levels of resistance have been compromising clinical treatment with conventional strategies (Miller et al., 2014; Muller et al., 2015).

A phage cocktail consisting of the two phages EFDG1 and EFLK1 (*Myoviridae*), was used in combination with ampicillin for treatment of septic peritonitis in a mouse model with the vancomycin resistant *E. faecalis* (VRE) strain V583, also referred to as ATCC 700802 (Gelman et al., 2018). Sub-optimal concentrations of the antibiotic were used, in order to mimic the PAS-effect. As a result, mouse mortality rates were similar between the dual therapy and single application of phages, but expectedly lower than the antibiotic-alone approach. The bacterial load in intra- and extra abdominal organs, such as liver and heart, was stronger reduced with the combined approach compared to either single therapy. Sensitivity to ampicillin, vancomycin, or to the phage cocktail of cultured bacteria from these organs revealed no difference between the single or dual treatments. Interestingly, recovery of active phages after the treatment revealed lower phage titers with the combined strategy compared to the phage-alone treatment. This result opposes to, what occurs in PAS, in which the antibiotic stimulates the phage production. Therefore, since the combined approach was more successful in reducing the bacterial load, positive antibiotic-phage interactions other than PAS must have determined the clinical outcome. Notably, phage treatment did not lead to an alteration of the gut microbiome as revealed by 16S rRNA amplicon sequencing of mice stool samples. This is valuable ancillary information considering that the potential impact of phage therapy on the natural microflora is poorly understood so far.

## Burkholderia cepacia

*B. cepacia* is an opportunistic pathogen responsible for rare cases of nosocomial infections and is especially related to pulmonary infections in cystic fibrosis patients. The symptoms of *B. cepacia* infections can differ from asymptomatic to respiratory failure and the treatment is problematic considering that this bacteria has an intrinsic resistance to many antibiotics (Horsley et al., 2016; Garcia et al., 2018).

Kamal and Dennis investigated PAS among several antibiotics belonging to four different classes and two distinct phages KS12 (*Myoviridae*) and KS14 (*Myoviridae, P2*-*like virus*) (Kamal and Dennis, 2015). By comparing plaque diameter in two *B. cepacia strains* C6433 and K56-2, PAS was observed with minocycline, levofloxacin, ceftazidime, meropenem, ciprofloxacin, and tetracycline, of which the three latter compounds produced the strongest results. No PAS was seen with ampicillin, kanamycin, and piperacillin. Cell filamentation occurred under exposure to meropenem and ciprofloxacin, which is in keeping with previous observations that this altered cell morphology favors PAS (Comeau et al., 2007). However, obviously PAS can also occur without filamentation, as tetracycline led to cell clustering, which enabled phages to move across the increased cell surfaces thereby increasing the chance of contacting cell receptors (Kamal and Dennis, 2015).

PAS was further confirmed with phage KS12 and strain K56-2 based on growth/kill curves and using larvae of *Galleria mellonella* as infection model (Kamal and Dennis, 2015). Survival rates of larvae were significantly increased with low-dose of meropenem and phage compared to either single treatment. Thus, the functionality of PAS could again be confirmed *in vivo*. It is known that *B. cepacia* can hardly be cleared from the lungs of patients with cystic fibrosis, for among other reasons, because antibiotics poorly penetrate into the tenacious biofilm. Ironically and fortunately, this could, however, have a practical medical implication, because with low amounts of antibiotics arriving at the bacterial target, optimal conditions for PAS might be realized. It would therefore be a worthwhile endeavor to investigate the therapeutic value of a joint application of antibiotics and phages in humans suffering from cystic fibrosis.

## Concluding Remarks

As a quintessence from the studies described in this review the combined treatment with phage and antibiotic is generally well-appreciated. Better clearance of bacterial cells and reduced evolvement of phage or antibiotic resistance are the major advantages of the joint therapy. Positive interactions between phages and antibiotics against which the pathogen is *a priori* resistant, gives hope that combined treatments will also be successful against the worst case of pandrug-resistant “super bugs” (Magiorakos et al., 2012). Depending on the type of antibiotic and phage, PAS has frequently been observed (e.g., Ryan et al., 2012; Uchiyama et al., 2018). And although representing no ubiquitous mechanism, PAS worked in biofilms as well (Ryan et al., 2012) and first data have demonstrated its occurrence under *in vivo* conditions (Kamal and Dennis, 2015). However, apart from neutral effects, the opposite of PAS has also been observed. The underlying negative interactions between the antimicrobials seem to be, however, only transient and the phages are not further disturbed by the presence of the antibiotic at a later treatment stage (Torres-Barceló et al., 2018). Combination therapies might greatly benefit from the careful choice of dosing and from the time points at which either antimicrobial substance is administered. In future studies particular attention should be given to sequential application, as at least two studies with *P. aeruginosa* demonstrated an improved therapeutic effect, when the antibiotic were introduced after phages had already started to tackle the bacteria (Torres-Barceló et al., 2014; Chaudhry et al., 2017). The attempt to evade the phage attack apparently makes the pathogen more vulnerable toward certain antibiotics. This concept warrants further investigation for other pathogenic bacteria and may ultimately turn out to be superior over simultaneous applications in most cases.

Additional interesting insights of phage/antibiotic combinations have recently been obtained using *Pseudomonas fluorescens*, which is a rare human pathogen, as model organism. Some of the results corroborate and some contradict previous findings with pathogenic bacteria. First, using a combination of kanamycin and phage SBW25Φ2 robustly prevented resistance evolution in *P. fluorescens* strain SBW25, which was not seen with either antimicrobial alone and which is in line with previous findings (Zhang and Buckling, 2012). Second, the sequential addition of rifampicin and the phage SBW25Φ 2 was more effective at reducing *P. fluorescens* SBW25 populations than their simultaneous employment. This is in line with the aforementioned observations of successful sequential treatment, except that this time the antibiotic was given first, followed by the phage. Here, stress induced by rifampicin made the population less able to evolve resistance against the phage (Escobar-Páramo et al., 2012). Lastly, using the same bacteria/phage system, the opposite of PAS was demonstrated. Using sub-MICs of the antibiotic streptomycin (Sm) increased the rate of phage resistance evolution and caused extinction of the phage. The combination also enhanced the evolution of Sm resistance compared with Sm alone (Cairns et al., 2017). Since Sm is a known mutagen, higher mutation rates may have been responsible for this counterintuitive development. However, the data also show that general conclusions about the functionality of phage/antibiotic combinations are difficult to draw. Negative interference might be more common as assumed, and it is possible that such experimental outcomes in the laboratory are less frequently reported than the positive ones. Negative results, however, should be encouraged for publication (Levin, 2014), as it avoids that mistakes are repeated and as it fosters the successful search for suitable phage/antibiotic combinations.

In order to achieve ever more improvements with phage/antibiotic combinations, the key to ultimate success might probably be the use of tailored bio-engineered phages as adjuvants for antibiotics (e.g., Lu and Collins, 2009). However, although we are in the age of synthetic biology (Barbu et al., 2016), the reluctance toward using replicating entities for therapy may be complicated by general public concerns surrounding the use of genetically manipulated compounds within humans (Bawa and Anilakumar, 2013). Nevertheless, bio-engineered phages may become broadly used eventually in the future, but it can be anticipated, that more progress with natural (i.e., genetically unmodified) phages will also be made in the meantime.

We conclude with the note, that at present, there is still a large gap in knowledge regarding the precise mechanisms that drive the phage/antibiotic interactions. Therefore, for now, it remains difficult to predict the optimal combinations for a given bacterial pathogen. Nevertheless, the encouraging results obtained so far suggest that the continued experimentation with phage/antibiotic combinations is an endeavor, which likely will pay off in future as an ultimate and robust remedy against multi-drug resistant bacteria.

## Author Contributions

H-PH designed the research. TT and MJ performed the research. TT and MJ analyzed the data and TT, MJ, and H-PH wrote the paper.

### Conflict of Interest Statement

The authors declare that the research was conducted in the absence of any commercial or financial relationships that could be construed as a potential conflict of interest.
